# Bone mineral density changes in the graft after acetabular impaction bone grafting in primary and revision hip surgery: A 2-year prospective follow-up

**DOI:** 10.1080/17453674.2018.1460776

**Published:** 2018-04-11

**Authors:** Davey M J M Gerhardt, Enrico De Visser, Baudewijn W Hendrickx, Berend W Schreurs, Job L C Van Susante

**Affiliations:** 1 Department of Orthopedics, Rijnstate Hospital, Arnhem; 2 Department of Radiology and Nuclear Medicine, Rijnstate Hospital, Arnhem; 3 Department of Orthopedics, Radboud University Medical Center, Nijmegen, The Netherlands

## Abstract

**Background and purpose:**

Impaction bone grafting (IBG) is an established method in hip revision surgery to reconstruct loss of bone stock. There is limited knowledge concerning the actual bone remodelling process within the allograft. We investigated with repeated bone mineral density (BMD) measurements the biological process of bone remodelling in the allograft in vivo. We hypothesized that an initial decrease in BMD would be followed by an increase towards baseline values.

**Patients and methods:**

Dual-energy X-ray absorptiometry (DXA) was used to measure BMD values in 3 regions of interest (ROI) in 20 patients (average age at surgery 70 years, 11 males) after an acetabular reconstruction with IBG and a cemented cup. A postoperative DXA was used as baseline and DXA was repeated at 3 and 6 months and at 1 and 2 years. The Oxford Hip Score (OHS), the 12-Item Short Form Health Survey (SF12), and a 0 to 100 mm visual analogue scale (VAS) for pain and satisfaction were obtained simultaneously.

**Results:**

The overall mean BMD in the IBG regions increased significantly by 9% (95% CI 2–15) at 2 years’ follow-up. In the cranial ROI BMD increased 14% (CI 6–22), whereas the BMD in the medial and caudal ROI showed an increase of 10% (CI 1–18) and 4% (CI –6–16), respectively. The OHS, SF12-mental, and VAS for pain all improved statistically significantly 2 years after surgery, with a mean VAS for satisfaction of 77 (CI 63–90) out of 100 points. The SF12-physical showed non-significant improvement.

**Interpretation:**

The BMD in the allograft gradually increased after IBG for acetabular reconstruction arthroplasties, particularly in the cranial ROI. An initial decrease in the BMD was not encountered. These BMD changes, as proxy measurements for bone remodeling, may indicate progressive apposition of vital new host bone in the grafted area.

In revision hip arthroplasty (THA), acetabular bone loss can be managed by impacting allograft bone chips (Slooff et al. 1996). An adequately impacted bone graft (IBG) provides initial stability for the implanted prosthesis and facilitates bone remodelling (Bolland et al. 2007). With a stable IBG, revascularization and incorporation of the graft into the host skeleton is stimulated, a process known as “creeping substitution” or bone graft incorporation. The impacted allograft serves as a non-vital matrix facilitating ingrowth of vital host bone and as such the restoration of host bone stock. There is little knowledge or histological data available about the speed of this process (Oakes et al. 2006). In a goat model Schimmel et al. (1998) reported graft resorption, bone apposition, and remodelling into new trabecular bone without hardly any graft remnants after 24 weeks. A subsequent human case series by van der Donk et al. (2002) investigated 24 acetabular biopsy specimens collected at different time points up to 9 years. First the graft consisted of non-vascularized graft remnants; at 3 to 5 months postoperatively a transition from the avital graft towards newly incorporated host bone was visible through a revascularization front. By 6 months approximately 30% of the graft had been incorporated. In biopsies from 6 months’ up to 9 years’ follow-up 70% of the graft had been incorporated and trabecular bone had been formed. Besides these studies no in-vivo data are available on the early biological process of bone graft remodelling after acetabular IBG.

In earlier studies repeated dual energy X-ray absorptiometry (DXA) proved successful to monitor periprosthetic BMD changes after different types of hip arthroplasties (Smolders et al. 2010, Huang et al. 2013, Smolders et al. 2013, Lazarinis et al. 2014) and was also used to monitor the incorporation of bone graft after spinal fusion (Hagenmaier et al. 2013).

We performed a 2-year prospective study with repeated DXA measurements, specifically to monitor BMD changes in vivo within the graft, to gain further insight into the process of bone graft incorporation after acetabular IBG. We hypothezised an initial BMD decrease or demineralization to occur within the IBG up to 6 months postoperatively as result of graft resorption, followed by a steadily increase in BMD as new vital trabecular bone is established.

## Patients and methods

### Study design

This exploratory study was designed to evaluate the BMD changes in the impacted bone graft region after acetabular reconstruction surgery. From December 2013 to December 2014, 25 consecutive patients with a clinical suspicion of cup loosening in the presence of an expected contained acetabular bony defect on plain pelvic radiographs were engaged in this exploratory study. Patients mean age was 70 (9) years (Table 1). The indication for acetabular IBG was determined preoperatively based on standard pelvic radiographs and sometimes combined with pelvic computerized tomography (CT) scanning to assess the degree of acetabular bone deterioration. Patients were excluded in the case of a current infection of the hip joint or other sites, a hip fracture, if immunocompromised or taking immunosuppressive medication, with a history of medication for osteoporosis (i.e., bisphosphonates), or neoplasm. Besides plain radiographs additional CT scanning of the pelvis was available in 10 cases. 20 patients (9 men) of 25 patients were intraoperatively confirmed to have cavitary defects type 2 (according to AAOS Classification of Acetabular Bone Loss) (D’Antonio et al. 1989), which could be reconstructed with acetabular bone impaction grafting without the use of metal augments. In 5 patients adequate reconstruction of the defect required metal meshes to ensure containment of the acetabular defect; these were excluded from DXA follow-up since these meshes would interfere with BMD measurements.

**Table 1.** Clinical details of the 20 patients who received an acetabular reconstruction using bone impaction grafting

Factor	THA
Sex (women/men)	11/9
Mean BMI (SD)	27 (4)
Mean age at surgery in years (SD)	70 (9)
Diagnosis	
primary osteoarthritis with acetabular bone defects	2
revision for aseptic loosening	14
revision THA secondary to infection	3
revision of hemiarthroplasty	1
Mean blood loss in mL (SD)	558 (229)
Mean surgery time in minutes (SD)	103 (25)
Mean cup inclination (SD)	50 (9)
Median acetabular cup size in mm (range)	49 (44–52)

### Study population and follow-up

14 acetabular revisions with IBG were performed for cup loosening with osteolytic bony defects due to profound polyethy­lene (PE) wear and 3 cups were revised as part of a 2-stage revision procedure for infection, which was considered healed at the second stage. The remaining 3 cases consisted of 1 hemiarthroplasty patient with acetabular protrusion, which was converted towards a THA, and 2 primary THA: 1 with profound acetabular protrusio and a 1 with a large post-traumatic acetabular bony defect. Of the 17 cup revisions (11 uncemented and 6 cemented), in 8 cases the cup revision was combined with a cemented stem revision (Exeter Stem, Stryker, Newbury, UK). All patients reached 1-year follow-up. In 3 patients BMD could not be measured at 2 years. 2 patients refused the final follow-up because of other illness and 1 patient was re-revised after 15 months towards a dual mobility cup for recurrent dislocations; however, clinical outcome scores could be assessed in all patients at 2 years excluding the 1 patient who was revised.

### Surgical technique

All patients received the standard surgical procedure for revision hip arthroplasty with acetabular IBG through a posterolateral approach, performed by 3 experienced hip surgeons. Preoperative digital templating for implant positioning (Easyvision, Philips Medical Systems, Eindhoven, The Netherlands) was carried out in all patients. The bone impaction grafting technique used is described in detail by Schreurs et al. (1998). Briefly, via a posterolateral approach the acetabulum was exposed and in revision cases the failed component was removed. All existing cement and fibrous tissue was removed. The sclerotic acetabular wall was penetrated by 5–10 superficial drill holes using a 3 mm burr. The contained defect was packed and filled layer by layer with handmade bone chips (7 to 10 mm) from fresh frozen femoral head allografts using a rongeur. Subsequently, the acetabular socket was restored using incremental metal impactors and a metal hammer. Bone cement (40 grams) with broad spectrum antibiotics (COPAL gentamycin + clindamycin, Heraeus Holding, Hanau, Germany) was placed on top of the impacted bone graft and pressurized with a seal. Subsequently a cemented PE cup was implanted in all cases, aiming for a 2-mm-thick cement layer (Exeter Contemporary Flanged Cup, Stryker, Newbury, UK). Cup positioning was measured on the direct postoperative standard anterior–posterior pelvic radiograph. A median outer cup size of 49 mm (range 44–52) was implanted with a mean cup inclination of 50 (8) degrees. Patients received 24 hours of systemic cefazolin (preoperative 1 x 1 g, postoperative 2 x 1 g), NSAID (3 x 50 mg diclofenac) to prevent heterotopic ossification was given for 1 week and anticoagulation therapy for 3 months (nadroparin 2,850 IE daily). Mobilization was started 24 hours after surgery with partial weight bearing on crutches for 6 weeks.

### Bone densitometry

**Figure 1.**
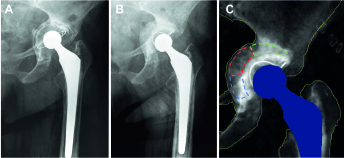
A: Example of an anterior–posterior (AP) hip radiograph with preoperative osteolytic bone deterioration with excessive cup protrusion and loosening. B: Postoperative AP radiograph with reconstruction of the contained acetabular bony defect using the bone impaction grafting technique without metal meshes. C: With dual-energy X-ray absorptiometry, bone mineral density was measured in 3 separate regions of interest covering the postoperative acetabular impacted bone graft: cranial (green), medial (red), and caudal (blue) to the polyethylene cup. The same ROI template was used for each subsequent time interval.

**Figure 2.**
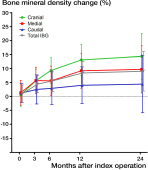
Point estimates of relative mean bone mineral density (BMD) changes within the impacted acetabular bone graft (g/cm^2^) compared with direct postoperative baseline values with 95% confidence intervals during 2 years’ follow-up. The total mean BMD (gray) is divided into 3 regions of interest: cranial (green), medial (red), and caudal (blue) to the acetabular cup.

**Figure 3.**
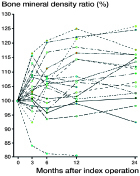
Spaghetti plot of measured bone mineral density (BMD) changes of the impacted acetabular bone graft (g/cm^2^) as percentage of the direct postoperative baseline values (%) at an individual patient level. Note: The outlier with a decrease in BMD of 20% corresponds with the early revision case due to recurrent dislocations of the hip.

The impacted bone graft BMD was measured with DXA (Lunar Prodigy, GE Healthcare, Little Chalfont, UK, software package Encore 2007, version 11.30.062). JvS identified and selected the impacted bone graft area on the postoperative DXA with reference to the intraoperative findings and preoperative radiographs and/or CT scan. Typically, a hemisphere surrounding the medial surface of the cup was selected with varying thickness depending on the volume of the grafted defect (Figure 1). This hemisphere or IBG area was divided into 3 regions (cranial, medial, and caudal) each covering approximately one-third of the surface. To enhance reproducibility, the defined template of the bone grafted area on the first postoperative DXA (Figure 1) was incorporated in each subsequent measurement by software recognizing the bony contours of the pelvis. Baseline measurements were performed within 2 weeks after surgery, at 3 and 6 months, and at 1 and 2 years. BMD values of the impacted bone graft obtained during the 2 years’ follow-up were compared with baseline levels (Table 2, see Supplementary data). BMD values are also expressed as a percentage against the original baseline levels (100%) (Table 2 and Figures 2 and 3). BMD values were categorized in a cranial, medial, and caudal ROI in relation to the acetabular cup. The software used in our study was designed to recognize the prosthesis and to measure periprosthetic acetabular BMD only (Figure 1). DXA scans and patient positioning were standardized according to a strict protocol; patients were positioned supine with their feet attached to a positioning device to obtain a reproducible 20° of internal rotation. A range of 15° internal to 15° external rotation yields a precision of 1.7% according to Mortimer et al. (1996). This precision error was also confirmed by a daily calibration procedure of the DXA where repeated measurements were performed on a phantom. In previous studies, regarding acetabular BMD changes after resurfacing and total hip arthroplasty, repeated measurements were also perfomed in study patients resulting in a mean coefficient of variation of 2.6% (SD 0.9) (Smolders et al. 2013); this was not repeated for the current study. Quality controls for the DXA equipment were undertaken daily according to the manufacturer’s guidelines to verify the stability of the system. No change was observed during the entire study period.

### Clinical outcome measurements

Clinical outcome measurements were completed preoperatively, at 3 and 6 months, and 1 and 2 years after surgery. This included the Oxford Hip Score (OHS), the 12-item Short Form Health Survey (SF12), a 0 (no pain) to 100 mm (maximum pain) visual analogue scale (VAS) for pain and a 0 (minimum) to 100 mm (maximum) VAS for satisfaction.

### Statistics

20 patients were included for 2-year follow-up in this exploratory study. This number of patients was selected and considered adequate to detect statistically significant differences regarding acetabular BMD changes based on earlier studies using this DXA technique (Smolders et al. 2010, Lazarinis et al. 2014). 20 patients have also proven to have adequate power monitoring BMD changes after bone grafting in a different field of interest, i.e., spinal fusion (Hagenmaier et al. 2013). For this reason, we performed no sample size calculation for the current study. All data were checked for normal distribution by means of the Shapiro–Wilk test. Normally distributed data are presented as mean (SD). Not-normal distributed data are presented as median (range). The absolute (g/cm^2^) and relative (%) BMD changes of each ROI over the observed period and clinical scores were compared with baseline values using linear mixed models with random intercept (patient) and random slope (time). Time (categorical) and sex were treated as fixed factors. Results from the mixed model were reported with use of point estimates with corresponding 95% confidence interval (CI). The assumptions for this model were checked and found to be adequately met. No adjustments for multiple testing were performed. Missing data were assumed to be missing at random; residuals of the model were normally distributed. Data of patients lost to follow-up were included up to their last measurement. Differences were considered statistically significant with a p-value <0.05. All statistical analyses were performed using SPSS software version 21.0 (IBM Corp, Armonk, NY, USA).

### Ethics, registration, funding, and potential conflicts of interest

Approval from the regional ethics committee from the Radboud University Nijmegen Medical Centre was obtained (NL 46305.091.13). Written informed consent was obtained from all patients. The study was registered in the Clinical Trials registry (NCT02061904). Research funding was obtained from Rijnstate Vriendenfonds. There are no conflicts of interest to be reported by any of the authors.

## Results

### Bone mineral density

The point estimates of the mean absolute (g/cm^2^) and relative (%) BMD values of the IBG are summarized in Table 2 (see Supplementary data) and are visualized in Figure 2. In the cranial region a higher BMD was measured at baseline compared with the medial (p = 0.02) and caudal regions (p = 0.07). In the overall grafted area, a BMD of 2.4 g/cm^2^ was measured at baseline. From postoperative baseline (100%) a gradual BMD increase of 9% was measured at 2 years’ follow-up for the overall grafted area. An initial decrease in BMD early after surgery, as hypothesized, was not seen. As for each specific ROI, the cranial region revealed the most pronounced BMD increase of 14%. In the medial region BMD increased 10% at 2 years’ follow-up, whereas for the caudal region this was 4%. Trends in measured BMD change for the grafted area at an individual patient level are summarized in Figure 3. Most patients reveal an increase in BMD over time, and some patients fluctuate around baseline levels. 1 outlier was seen with a decrease in BMD close to 20% at 1-year follow-up. This patient was the single revision case at 15 months for recurrent (4 times) dislocation. Figure 2 represents mean values (%) for the entire group and as such the overall trend in BMD change is clearly visible (Figure 4 and Table 3, see Supplementary data).

### Clinical outcome

All clinical outcome scores had improved at 2-year follow-up (Table 3, see Supplementary data).

## Discussion

There is a knowledge gap regarding the actual bone remodelling process of impacted bone grafts used in acetabular reconstruction surgery. This prospective DXA study was designed to measure BMD changes after an acetabular reconstruction with impacted bone grafting, monitoring the process of acetabular bone graft incorporation in vivo. The expected BMD decrease in the first 6 months after surgery, as observed in studies regarding acetabular BMD changes after primary hip arthroplasty, was not seen (Digas et al. 2006, Smolders et al. 2013, Lazarinis et al. 2014). In contrast to our hypothesis, BMD gradually increased directly from postoperative baseline levels. An increasing trend was seen in all 3 separate ROIs with the strongest increase of 14% cranial to the cup. In addition, baseline BMD levels (g/cm2) were significantly higher in this cranial region immediately after surgery compared with the medial and caudal regions. These higher direct postoperative BMD values correspond with the direction of impaction force at the time of implantation and may thus represent the presence of denser bone graft. The encountered BMD increase up to 2 years after surgery may be indicative of the gradual apposition of vital new bone whereas initial bone depletion and excessive loss of density of the graft does not seem to occur. Interestingly, as noted in Figure 2, 3 patients do show minor BMD changes or even a slight decrease in BMD within the IBG. This is an interesting result, on top of which 1 outlier is seen with a decrease in BMD close to 20% at 1 year-follow-up. This outlier experienced recurrent hip dislocations and the inability of full weight-bearing may have affected the process of graft incorporation in this specific case, resulting in a steep BMD decrease.

The functional outcome scores all improved significantly after surgery and patients reported a high satisfaction rate in accordance with earlier literature (Arumugam et al. 2015, Te Stroet et al. 2015b).

### Bone remodelling after IBG

The impaction bone grafting technique has already proven its value in hip arthroplasty since it was introduced (Slooff et al. 1984). With this biological approach in reconstructing the acetabulum, satisfying long-term results on implant survival and preservation of the bone stock have been published ­(Schreurs et al. 2004, Comba et al. 2006, Schreurs et al. 2009, Te Stroet et al. 2015a). The process of bone graft incorporation depends on the initial mechanical stability of the IBG and the biological interaction with the host bone (Giesen et al. 1999). The bone graft resorption and bone ingrowth should be well balanced in order to retain its mechanical stability and to prevent cup migration, as confirmed by our findings as we did not observe a BMD decrease.

### BMD measurements after IBG

Limited data are available regarding the process of acetabular bone graft incorporation with special reference to its speed and correlation with BMD changes (Buma et al. 1996, Ullmark and Obrant 2002, van der Donk et al. 2002). The available literature so far concerns BMD changes after IBG on the femoral side (Nesse et al. 2003, Grochola et al. 2008). The groups of Ullmark et al. (2009) and Piert et al. (1999) have reported on the use of positron emission tomography (PET) in monitoring bone metabolism within the IBG. By measuring the uptake of [18F]fluoride in various regions of the IBG this active process of bone graft incorporation could be quantified. In a small cohort of 7 cup revisions with segmental and cavitary defects reconstructed with IBG, an increased uptake of [18F]fluoride was measured up to 4 months after surgery compared with the uptake measured in the healthy contralateral hip (Ullmark et al. 2009). Subsequently normalizing uptake levels were measured at 1 year postoperatively, indicating the IBG having been transformed to living bone stock, similar to our results.

### Strength and limitations

The strength of this study is that BMD has prospectively been monitored in a consecutive series of patients with simple cavitary defects reconstructed with IBG. There are some limitations. First, we included only 20 patients with varying size and volume of acetabular defects. On the other hand, this is the reality in clinical practice and the observed mean trend in BMD changes appeared to overlap globally with the curves for each individual patient (Figure 3). We do not believe that a larger number of patients or standardization of the defects would have given a different outcome. However, future studies with a larger number of patients may allow further insight into the differences in BMD changes in different ROI, as our study clearly indicates a more pronounced BMD increase in the cranial (weightbearing) area. Second, CT scanning could also have been considered to monitor BMD changes over time. CT scanning may be more accurate, evaluating true bone mineral density alterations specifically within the IBG in a 3-dimensional manner, thus excluding over-projection of cortical bone at the acetabular rim, which is inevitable with two-dimensional DXA measurements. On the other hand, CT scanning also generates significantly higher radiation doses and costs and the bias from concomitant BMD change in the cortical walls is expected to be minimal. In our opinion, at this exploraory stage of evaluating the process of bone remodeling in IBG, DXA is useful due to its proven reproducible nature, low radiation dose, and costs. Finally, template selection for the DXA measurements was performed by a single individual and could not be standardized. All defects were contained and, in each case, the inner surface of the acetabular component thus had to be surrounded by a layer of bone graft, which area appeared to be recognizable on the first postoperative DXA. Only the size and volume of the defect could differ between patients, which resulted in differing thickness of the bone graft layer. Defining the grafted area on the first DXA scan together with the subsequent division in a cranial, medial, and caudal graft area had to be individualized. Since we used a conservative approach where the template was chosen within the boundaries of the grafted area and the cup cement mantle this limitation is of little importance. Also, the same template was used in all subsequent DXA measurements.

### Summary

A gradual increase in BMD within the grafted area, particularly in the cranial ROI, was encountered up to 2 years after surgery. In contrast to our hypothesis, there was no initial decrease in BMD. The profound BMD decrease in the one early revision case supports our belief that early weightbearing is important for adequate incorporation of the graft in at least the cavitary defects.

### Supplementary data

Tables 2 and 3, and Figure 4 are available as supplementary data in the online version of this article, http://dx.doi.org/10.1080/17453674.2018.1460776

DG: Local investigator and PhD student on bone mineral density changes after hip arthroplasty. EdV: manuscript review. BH: responsible for all the patients’ DXA follow-up. BS: manuscript review. JvS: Principle investigator, study design, writing manuscript.

*Acta* thanks Stergios Lazarinis and Gösta Ullmark for help with peer review of this study.

## Supplementary Material

IORT_A_1460776_SUPP.pdf
