# Supplementary material for: Bone mineral density changes in the graft after acetabular impaction bone grafting in primary and revision hip surgery: A 2-year prospective follow-up
Source: Acta Orthop. 2018 Apr 11;89(3):302–7. doi: 10.1080/17453674.2018.1460776 (PMC6055772; doi:10.1080/17453674.2018.1460776)
Supplement: IORT_A_1460776_SUPP.pdf [file IORT_A_1460776_SM9126.pdf]

Supplementary data

Table 2. Point estimates of mean bone mineral density (g/cm^2) and as percentage of baseline values (100%) with 95% confidence intervals per region of interest of the impacted bone graft at each study interval [a]

Region	0 months n = 20	3 months n = 20	6 months n = 20	1 year n = 20	2 years n = 17
Cranial, g/cm^2	2.5 (2.1–2.8)	2.6 [b] (2.3–3.0)	2.7 [b] (2.3–3.0)	2.8 [b] (2.4–3.0)	2.8 [b] (2.5–3.1)
%	100 (97–104)	+6 [b] (2–10)	+9 [b] (5–13)	+13 [b] (8–18)	+14 [b] (6–22)
Medial, g/cm^2	2.3 (2.0–2.7)	2.4 (2.1–2.8)	2.4 [b] (2.1–2.7)	2.5 [b] (2.2–2.8)	2.5 [b] (2.1–2.9)
%	101 (97–106)	+6 [b] (1–11)	+6 [b] (0–11)	+9 [b] (3–16)	10 [b] (1–18)
Caudal, g/cm^2	2.3 (2.0–2.6)	2.3 (2.0–2.7)	2.3 (1.9–2.7)	2.3 (1.9–2.7)	2.4 (2.0–2.7)
%	101 (97–106)	+3 (–3–8)	+3 (–3–9)	+4 (–3–11)	+4 (–6–16)
Total, g/cm^2	2.4 (2.1–2.6)	2.4 [b] (2.2–2.7)	2.5 [b] (2.2–2.8)	2.5 [b] (2.3–2.9)	2.6 [b] (2.2–2.9)
%	101 (98–104)	+4 [b] (1–8)	+6 [b] (2–10)	+8 [b] (4–13)	+9 [b] (2–15)

[a] BMD changes were tested for significant difference compared with the postoperative value in a linear mixed model.
[b] Statistically significant different BMD value compared with the baseline value.

Table 3. Point estimates of clinical scores over time with 95% confidence interval [a]

Type	Preoperative n = 20	3 months n = 20	6 months n = 20	1 year n = 20	2 years n = 19
Oxford Hip Score	39 (35–43)	31 [b] (27–35)	27 [b] (23–32)	26 [b] (21–31)	25 [b] (17–34)
SF 12-physical	46 (34–58)	40 (28–51)	51 (40–64)	50 (37–63)	50 (34–67)
SF12-mental	50 (38–58)	49 (40–61)	61 [b] (49–72)	58 [b] (46–70)	63 [b] (49–78)
VAS-pain	60 (46–71)	35 [b] (25–48)	29 [b] (19–40)	29 [b] (17–42)	31 [b] (19–48)
VAS-satisfaction	–	71 (59–82)	78 (65–89)	77 (65–88)	77 (63–90)

[a] Results were tested for significant difference compared with the postoperative value in a linear mixed model. Oxford Hip Score (OHS), SF12-score, and a visual analogue scale (VAS) for pain and satisfaction: “0” is minimum score and “100” is maximum score.
[b] Statistically significant different BMD value compared with the baseline value.

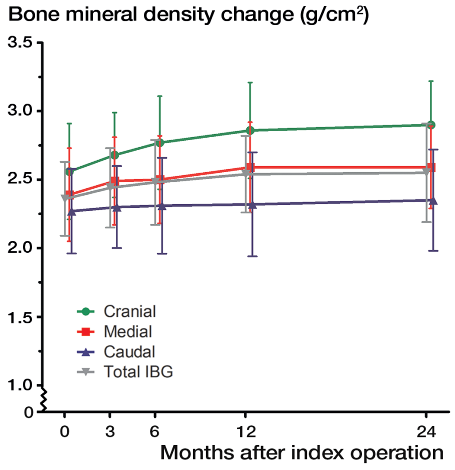

Figure 4. Point estimates of mean bone mineral density (BMD) changes of the impacted bone graft (g/cm^2) with 95% confidence intervals during 2 years’ follow-up. The mean total BMD (grey) is divided into 3 regions of interest: cranial (green), medial (red), and caudal (blue) to the acetabular cup.
